# Modeling the prevention of colorectal cancer from the combined impact of host and behavioral risk factors

**DOI:** 10.1038/gim.2016.101

**Published:** 2016-08-04

**Authors:** Matthew Frampton, Richard S. Houlston

**Affiliations:** 1 The Centre for Molecular Pathology, The Royal Marsden NHS Foundation Trust, London, UK; 2 Division of Genetics and Epidemiology, The Institute of Cancer Research, London, UK; 3 Division of Molecular Pathology, The Institute of Cancer Research, London, UK

**Keywords:** colorectal cancer, genetic, lifestyle, polygenic risk, prevention

## Abstract

**Purpose:**

This study investigated the utility of modeling modifiable lifestyle risk factors in addition to genetic variation in colorectal cancer (CRC) screening/prevention.

**Methods:**

We derived a polygenic risk score for CRC susceptibility variants in combination with the established nongenetic risk factors of inflammatory bowel disease (IBD), adiposity, alcohol, red meat, fruit, vegetables, smoking, physical activity, and aspirin. We used the 37 known risk variants and 50 and 100% of all risk variants as calculated from a heritability estimate. We derived absolute risk from UK population age structure, incidence, and mortality rate data.

**Results:**

Taking into account all risk factors (known variants), 42.2% of 55- to 59-year-old men with CRC have a risk at least as high as that of an average 60**-**year-old, the minimum eligible age for the UK NHS National Bowel Cancer Screening Program. If the male population is stratified by known variants and IBD status, then risk-difference estimates imply that for 10,000 50-year-old men in the 99th percentile, 760 cases could be prevented over a 25-year period through the modifiable risk factors, but in the lowest percentile, only 90 could be prevented.

**Conclusion:**

CRC screening and prevention centered on modifiable risk factors could be optimized if targeted at individuals at higher polygenic risk.

*Genet Med* **19** 3, 314–321.

## Introduction

Colorectal cancer (CRC) is a significant public health issue in developed countries.^1^ Despite advances in the clinical management of CRC, 5-year patient survival is typically only approximately 55%.^2^ This poor prognosis and the increasing incidence rates seen in Western countries^1^ have provided strong motivation for establishing population screening programs for early detection of CRC.^3,4^ Additionally, there is increasing interest in developing and implementing strategies to lessen the risk of developing CRC. The effectiveness of screening and prevention programs is likely to be optimized if directed toward those identified to be at highest risk for CRC. However, to date, methods for predicting the individuals in the population who are at increased risk and in whom targeted prevention can be directed have been relatively limited.

CRC has a strong heritable basis, and an increasing number of susceptibility loci for CRC are now being discovered through genome-wide association studies (GWAS).^5,6,7,8,9,10,11,12,13,14,15,16,17,18^ So far, these studies have identified single-nucleotide polymorphisms (SNPs) at 37 loci that are robustly associated with CRC risk.^19^ Although individually these variants have only a modest impact on CRC, the combined effect of multiple SNPs has the potential to provide useful levels of risk stratification in the population.^19,20,21,22^

We have recently examined the value of genetic information in the context of population screening programs for the early detection of CRC by deriving polygenic risk scores (PRS) for the known CRC risk SNPs.^19^ However, there are several well-established modifiable risk factors for CRC, and building predictive models incorporating both genetic and nongenetic risk factors should enable a more complete assessment of CRC risk. Importantly, such a model affords an opportunity to target risk modification through lifestyle change programs in addition to screening and chemoprevention.

Here, we examined the potential impact of genetic information in combination with independent modifiable risk factors for CRC prevention by implementing a model-based analysis. We also assessed the probable impact of using aspirin as chemo-prevention in the context of a prevention program using stratified CRC risk. Because CRC rates are higher in males, we confined our analysis to men acknowledging that similar conclusions are likely to also apply to women, albeit perhaps with less significant impact. This is due primarily to lower CRC rates in females, and also to lower risk attributable to the modifiable risk factors, thus probably providing slightly less scope for risk reduction via such prevention programs.

## Materials and Methods

### Calculation of polygenic risk score

We used the published allele frequencies and per-allele relative risk (RR) using a log-additive model of interaction between risk alleles (**Supplementary Table S1** online) to estimate the parameters of the normal distribution of PRS. If *Ι* corresponds to the total number of risk alleles, *β* corresponds to the log odds ratio (OR) of a risk allele, and *p* corresponds to the risk allele frequency, then *μ* and *σ *^*2*^ are given by:


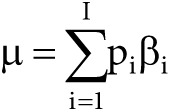



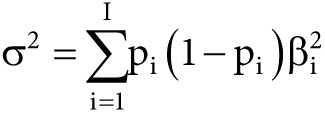


Hence, the distribution of risk on an RR scale in the population is log-normal with mean *μ* and variance *σ *^*2*^. The distribution of PRS for cases is displaced to the right by *σ *^*2*^; therefore, the population *μ* can be set to *−σ *^*2*^*/2* to give a mean RR in the population of unity (PRS = 0).

To derive the minimum number of risk alleles that an individual requires to equal or exceed a certain PRS threshold (e.g., to be in the top 1%), the alleles are first sorted into descending order according to the difference between the log OR and 0 (i.e., an RR of 1.0). If there are *Ι* risk alleles, *j* ≤ *Ι*, *r*_*i*_ = log risk OR of *i*^*th*^ allele (0 in the case of a protective allele), *n*_*i*_ = log nonrisk OR of *i*^*th*^ allele (negative in the case of a protective allele), and *PRS*_*thresh*_ = *PRS* threshold, then the minimum number *i* is:


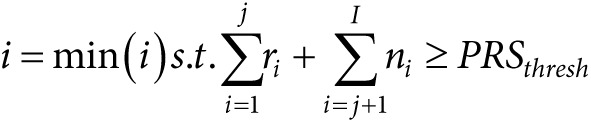


The method for deriving the maximum number is identical except that the alleles are first sorted into ascending, not descending, order.

There is an overrepresentation of association signals in existing GWAS after accounting for known risk SNPs, so additional risk variants should be identifiable by new GWAS. Using genome-wide complex trait analysis,^23^ we previously estimated the heritability of CRC for all common variation to be 19% (±8%),^19^ which translates to this class of susceptibility accounting for 55% of the familial risk. The inclusion of these as-yet-unidentified risk variants in future models will inevitably increase the precision of PRS-based personalized risk profiling for CRC. To address this prospect, in addition to evaluating the utility of PRS based on the 37 SNPs documented to influence CRC risk in Europeans, we also used the genome-wide complex trait analysis heritability estimate to examine model predictive values assuming that 50 and 100% of all common risk variants have been identified.

To examine the extent by which polygenic risk information from SNP genotype can stratify CRC by itself or in combination with modifiable risk factors, we estimated the expected RR distribution and corresponding absolute risks of CRC by incorporating various sets of risk factors for the UK population. We assessed the discriminatory capability of all models by calculating the area under the curve (AUC) for the receiver-operator characteristic curve.

### Nongenetic risk factors

Epidemiological studies have implicated a number of nongenetic factors as determinants of CRC risk, notably alcohol consumption, adiposity, red meat consumption, smoking, fruit and vegetable consumption, and lack of physical activity. Additional factors influencing CRC risk include aspirin/nonsteroidal anti-inflammatory drug use and inflammatory bowel disease (IBD).^24^ Our risk models incorporate all of these risk factors and use body mass index (BMI) as a measure of adiposity. The nongenetic risk factors are included in a risk model in the same way as the risk alleles. In these summations for mean and variance, for a nongenetic risk factor, *β* is the log OR for a level/category and *p* is the proportion of the population that is exposed; hence, whereas a SNP contributes two terms (one for each allele), a nongenetic risk factor contributes *n*, where *n* is the number of levels/categories.

We derived ORs for each of the risk factors from recent meta-analyses that only considered studies of European ancestry and that accounted for the presence of other risk factors to minimize any confounding.^24,25^ For alcohol consumption, the reported RR for CRC was 1.06 per five drinks per week, for BMI it was 1.29 per 8-kg/m^2^ increase over normal/low BMI (i.e., ≤25 kg/m^2^), for red meat consumption it was 1.12 for five servings per week compared to none, for fruit consumption it was 0.84 for three servings per day compared to none, for vegetable consumption it was 0.86 for five servings per day compared to none, for physical activity it was 0.88 for a twofold increase in the standardized score, and for IBD (ulcerative colitis and/or Crohn disease) it was a 2.93-fold increase compared to not having IBD. The risk of CRC for ever-smokers is reported to be 1.18 when compared to never-smokers.^25^

The reported prevalence of IBD in the United Kingdom is 4 out of 10,000 of the population in recent surveys.^26^ The exposure levels in the general population for alcohol, BMI, red meat, fruit, vegetables, physical activity, and smoking were drawn from the most recent available year in the Health Survey for England (HSE),^27,28^ which was 2008 for red meat consumption and 2013 for other risk factors (**Supplementary Materials and Methods** online). Incorporating each factor into our model required the degree of exposure in the general population to align with the risk factor levels in the meta-analysis. This was true for smoking, but not for the others; therefore, we fitted continuous probability distributions to the censored HSE data using the R package *fitdistrplus*. A normal distribution was appropriate for BMI but not for the other risk factors, which have right-tailed distributions. For these, we fitted gamma distributions because they provided a superior fit compared to alternatives (exponential, log-normal, log-logistic, Weibull and Gumbell) when assessed by Bayesian information criterion, Akaike information criterion, or log likelihood. For physical activity, we used the parameters of its fitted gamma distribution to calculate standard scores. When the data included a 0 interval (e.g., individuals who consume no alcohol), it was necessary to apply distribution shifting to fit certain distributions. Hence, a small increment was made to the left and right ends of each interval prior to fitting, which was then subtracted postfitting.

Obtaining reliable data on aspirin usage in the general population in England is inherently problematic because the HSE contains data on prescribed usage but not all usage. Therefore, for the purposes of this modeling exercise, we made use of data from a large US cohort study^29^ that examined associations between long-term (≥5 years) daily use of adult-strength aspirin (≥325 mg/day) and incidence of 10 cancers in 69,810 men within the Cancer Prevention Study II Nutrition Cohort. At enrollment, 2.3% of men reported long-term daily usage, which increased to 4.6% during the follow-up period. After adjustment for other risk factors, long-term daily aspirin use was associated with a significant 32% reduction in CRC (RR = 0.68; 95% CI, 0.52–0.90).

Therefore, we initially developed six PRS-based risk models: model 1, based on the known risk SNPs; model 2, based on the known risk SNPs plus IBD and the modifiable risk factors of alcohol consumption, BMI, red meat consumption, smoking, consumption of fruits and vegetables, physical activity, and aspirin usage; models 3 and 4, which are equivalent to models 1 and 2 but assume half of all risk variants are known; and models 5 and 6, which are again equivalent to models 1 and 2 but assume all risk variants are known.

We also used risk models that incorporate only the host risk factors of known risk SNPs and IBD to investigate the utility of polygenic risk in targeting CRC prevention programs that are centered on the modifiable risk factors. In such a risk model, every individual is effectively assigned average risk attributable to the modifiable risk factors (RR = 1.0). The risk score for an individual in the *X*^*th*^ percentile, *RS*_*X*_, with a healthy (unhealthy) lifestyle can be calculated by adding the difference between the average and minimum (maximum) risk score attributable to the modifiable risk factors. If there are a total of *Ι* levels/categories of modifiable risk factors, then *RS*_*modav*_, *RS*_*modmin*_, and *RS*_*modmax*_, the average, minimum, and maximum risk scores for the modifiable risk factors, are:


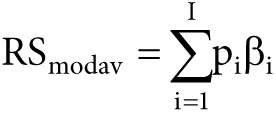



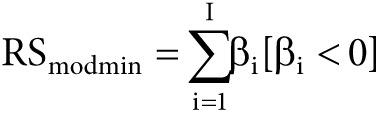



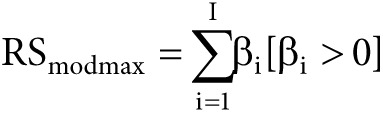


Thus, the risk scores for an individual in the *X*^*th*^ percentile with a healthy lifestyle and an individual with an unhealthy lifestyle are:


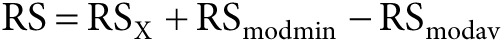



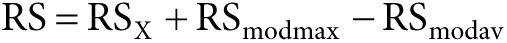


The RR compared to an individual in the 50th percentile with an average lifestyle is:


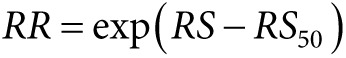


### Calculation of absolute CRC risks

We obtained incident CRC registrations, deaths from CRC and all causes, and mid-year population estimates in 1-year age bands for England (2001–2012) from the Office for National Statistics. Averaging incidence and mortality rates for this period, we used DevCan 6.4.1 software^24^ to derive the baseline age-conditional absolute risk of CRC. From this and RR estimates, we derived the age-conditional absolute CRC risk for individuals.

## Results

### Utility of polygenic risk in colorectal cancer risk stratification

**Figure 1:**
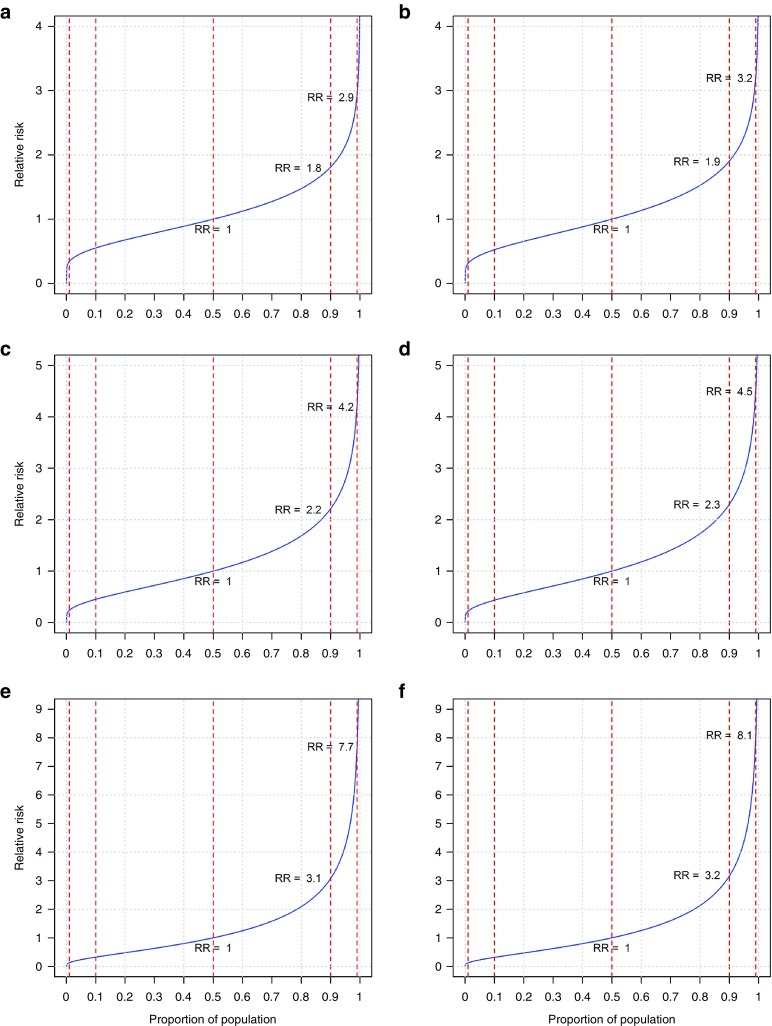
**Population distribution of colorectal cancer relative risk scores (compared to population median risk) in the six models.** The blue line corresponds to the distribution of relative risk (RR) across the population; the red lines correspond to the 1st, 10th, 50th, 90th, and 99th percentiles. The RR figures presented in black are the averages in the (i) 50th, (ii) 90th, and (iii) 99th percentiles of genetic risk.

The 37 currently identified susceptibility variants for CRC confer a polygenic variance of 0.21 and account for approximately 14.5% of the familial risk of CRC. **Figure 1a** shows the PRS for CRC based only on the known risk SNPs (model 1). Here, individuals in the top 10% of CRC risk have a 1.8-fold higher risk of CRC compared to the population median, and those within the top 1% (i.e., 27–54 risk alleles) have a 2.9-fold increased risk. **Figure 1b** shows risk after inclusion of IBD and the modifiable lifestyle risk factors (model 2). Now, individuals within the top 10% and 1% show a 1.9-fold and 3.2-fold increased risk, respectively. Similarly, when using model 4 instead of model 3 (**Figure 1c**,**d**), RR increases from 2.2 to 2.3 for the top 10%, and from 4.2 to 4.5 for the top 1%. When using model 6 instead of model 5 (**Figure 1e**,**f**), RR increases from 3.1 to 3.2 for the top 10%, and from 7.7 to 8.1 for the top 1%.

**Table 1** shows the receiver-operator characteristic AUC metric for each of the six models. Inclusion of IBD and the modifiable risk factors leads to a small increase from 0.63 to 0.64 when using known risk SNPs (models 1 and 2), from 0.67 to 0.68 when assuming half of risk SNPs are known (models 3 and 4), and from 0.73 to 0.74 when assuming all risk SNPs are known (models 5 and 6).

Individuals are eligible for the UK NHS national bowel cancer screening program at age 60 (ref. 3). The effectiveness of such screening and prevention programs will be improved if targeted toward individuals who are at the highest risk for CRC, including younger individuals at high risk. In England during the period from 2001 to 2012 inclusive, an average of 1,401,447 of the male population was aged 55–59 and 1,264 were diagnosed with CRC. **Table 1** shows the percentage of this population for which the six models identify as being at higher risk for CRC. For the purposes of this comparison, higher risk is defined as a 10-year absolute risk of developing CRC greater than or equal to that of an average 60-year-old man, which is 1.96%. **Table 1** shows the six models identifying approximately 22 to 24% of the population as being at higher risk. Inclusion of IBD and the modifiable risk factors increases the percentage of cases identified as being at higher risk, for example, from 40.1 to 42.2% when using the known risk SNPs (models 1 and 2). Model 6 identifies 57.7% of cases as higher risk.

### Utility of polygenic risk in targeting colorectal cancer prevention

**Figure 2:**
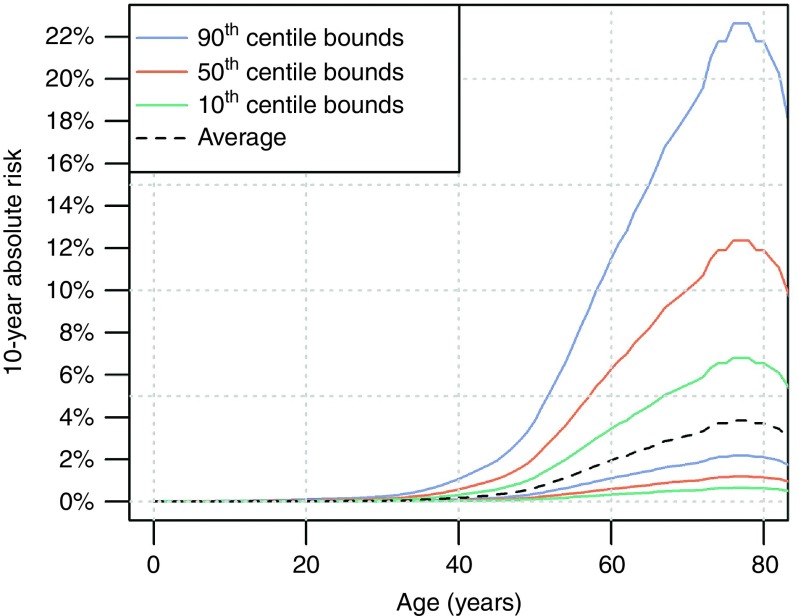
**The 10-year absolute risk of being diagnosed with colorectal cancer in men (England, 2001–2012) in the 10th, 50th, and 90th percentiles of a risk model that incorporates common genetic variation (known risk single-nucleotide polymorphisms) and inflammatory bowel disease and where the lower and upper bounds are defined by modifiable lifestyle risk factors.** Lower and upper bounds for modifiable risk factors are: alcohol, <5 and ≥50 drinks per week; body mass index, <33 kg/m^2^ and ≥41 kg/m^2^; red meat consumption, <5 and ≥5 servings per week; fruit consumption, ≥3 and <3 servings per day; vegetable consumption, ≥5 and <5 servings per day; physical activity, ≥4 and <2 standard deviations above the mean number of hours per day; smoking, never and ever; and aspirin, long-term use and no long-term use.

In **Figure 2**, we considered men in the 10th, 50th, and 90th percentiles of a risk model defined by the host risk factors of common genetic variation and IBD and their 10-year risk of CRC according to modifiable risk factor status. The bounds were calculated using the most extreme levels of the modifiable risk factors that have nonzero counts in our data sources. This figure shows that the 10-year risk of CRC can vary substantially depending on the number of modifiable lifestyle risk factors. For example, a 60-year-old man in the 90th percentile of risk according to genetics (known risk SNPs) with IBD can have a 10-year risk ranging from 1 to 11%. **Figure 2** also shows that, depending on lifestyle, it is possible for an individual in the 90th percentile to have a lower risk than average and for an individual in the 10th percentile to have a higher risk than average.

**Table 2** shows the 25-year risk of developing CRC for a 50-year-old man in England, where the population is again stratified by the host risk factors. We chose to base calculations on the 25-year risk because the UK Scottish bowel cancer screening program is open to those aged 50–74 (ref. 4). **Table 2** shows the estimated risk reductions achieved by minimizing risk attributable to the modifiable risk factors (i.e., by setting the modifiable risk factor levels to those used in calculating the lower bounds in **Figure 2**). These risk-difference estimates imply that prevention programs involving the modifiable risk factors could be optimized if targeted at individuals who are at higher risk according to the host risk factors. For example, the risk-difference estimates in **Table 2** indicate that if 10,000 men in the lowest and highest percentiles undertook long-term aspirin usage, then 40 and 340 cases of CRC, respectively, would be prevented over a 25-year period. If they did not undertake long-term aspirin usage but minimized risk with respect to the other modifiable risk factors, then 70 and 610 cases of CRC would be prevented. If they undertook long-term aspirin usage as well, then 90 and 760 cases would be prevented. Assuming half of risk SNPs are known, if all 10,000 men in the lowest and highest percentiles minimized risk with respect to all modifiable risk factors (including undertaking long-term aspirin use), then 60 and 1,090 cases, respectively, would be prevented. If all risk SNPs are known, then the number of cases prevented would be 30 and 1,980, respectively.

## Discussion

Our findings serve to illustrate how information on polygenic risk, in conjunction with modifiable risk factors, might be used in primary prevention to identify subgroups of the population at increased risk for CRC and who are most likely to benefit from advice or intervention. Conventionally, the discriminatory accuracy of risk prediction models is assessed using receiver-operator characteristic curves. However, the AUC depends solely on the RR parameter and, as is the case here, when the baseline risk is high even a modest increase in AUC has the potential to substantially enhance risk stratification of the population.

Here, we have not incorporated family history information on CRC because there is potential for recall bias and inaccuracy in the reporting of family history.^23,30^ Notably, surveys have often reported that CRC in relatives can be significantly under-reported.^30^ Profiling for CRC susceptibility through genetic testing directly addresses such shortcomings.

Although our analysis supports the tenet that the integration of genetic and nongenetic risk factor information can provide meaningful risk stratification, we acknowledge that there are a number of limitations to our model-based analysis. First, we relied on published RR estimates from a large meta-analysis and population-based distributions. Second, our analysis also relied on the key assumption that genetic and lifestyle risks act multiplicatively. Additionally, we assumed that the impact of a chemoprevention agent or improvement in lifestyle on CRC risk will be uniform across genotypes. Within the genetic component, few interactions of risk between genetic loci have been identified, so the assumption of independence is appropriate. For lifestyle risk factors, assumptions of independence are more problematic. Although we made use of RRs for each factor estimated in the presence of relevant covariates, levels of risk inflation can still be an issue if there are inaccuracies in the estimated population frequencies. Third, although there is currently little evidence for gene–environment interactions in CRC, further studies are required to evaluate this empirically and, if appropriate, estimate absolute risks for combinations of risk factors and interventions. However, very large studies are required to address this challenge because of the inevitable relative paucity of key data at extremes. Finally, the assessment of the performance of any model to provide predictions of risk needs to be calibrated in prospective cohort studies.

Accepting these caveats, a number of conclusions can be gleaned from our analysis, notably that modifying lifestyle risk factors would lead to significant changes in the proportion of the population at risk for CRC. Although this could have been predicted a priori by the higher ORs for these factors, it is the combination of RR together with the prevalence of the factor within the population that determines the overall impact. Population screening programs for the early detection of CRC are widespread.^3,4^ In contrast, there is currently a low profile for strategies targeting lifestyle factors in those at highest risk, which is particularly relevant for younger age groups. Such an approach could potentially encourage behavioral changes and help to significantly reduce CRC rates. This is especially important because over recent decades the lifetime risk of CRC has increased,^1^ owing to a change in the dietary and lifestyle factors of the general population, which is characterized by higher levels of obesity and more sedentary pastimes.^31^ However, research is required to determine how individuals might actually respond to risk assessment based on genetic information.

Although modeling indicates that certain individuals will reduce their CRC risk by changing their behavior, the time needed for changes in environmental risk factors to have an effect on risk is unknown, is likely to differ for each factor, and is something only able to be determined prospectively. Important research is needed to further elucidate the genetic and environmental contributions to risk and to measure the long-term impact of behavioral change on CRC outcomes. Finally, if profiling and risk stratification are to be adopted in public health, then prediction tools are needed that are easy to use by health-care professionals.

## Disclosure

The authors declare no conflict of interest.

## Figures and Tables

**Table 1:**
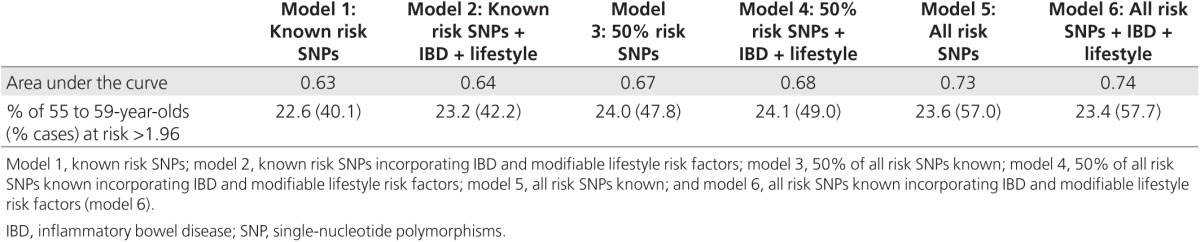
Area under the curve and identification of men aged 55–59 years in the population who are at increased risk for colorectal cancer (i.e., ≥1.96% 10-year absolute risk)

**Table 2:**
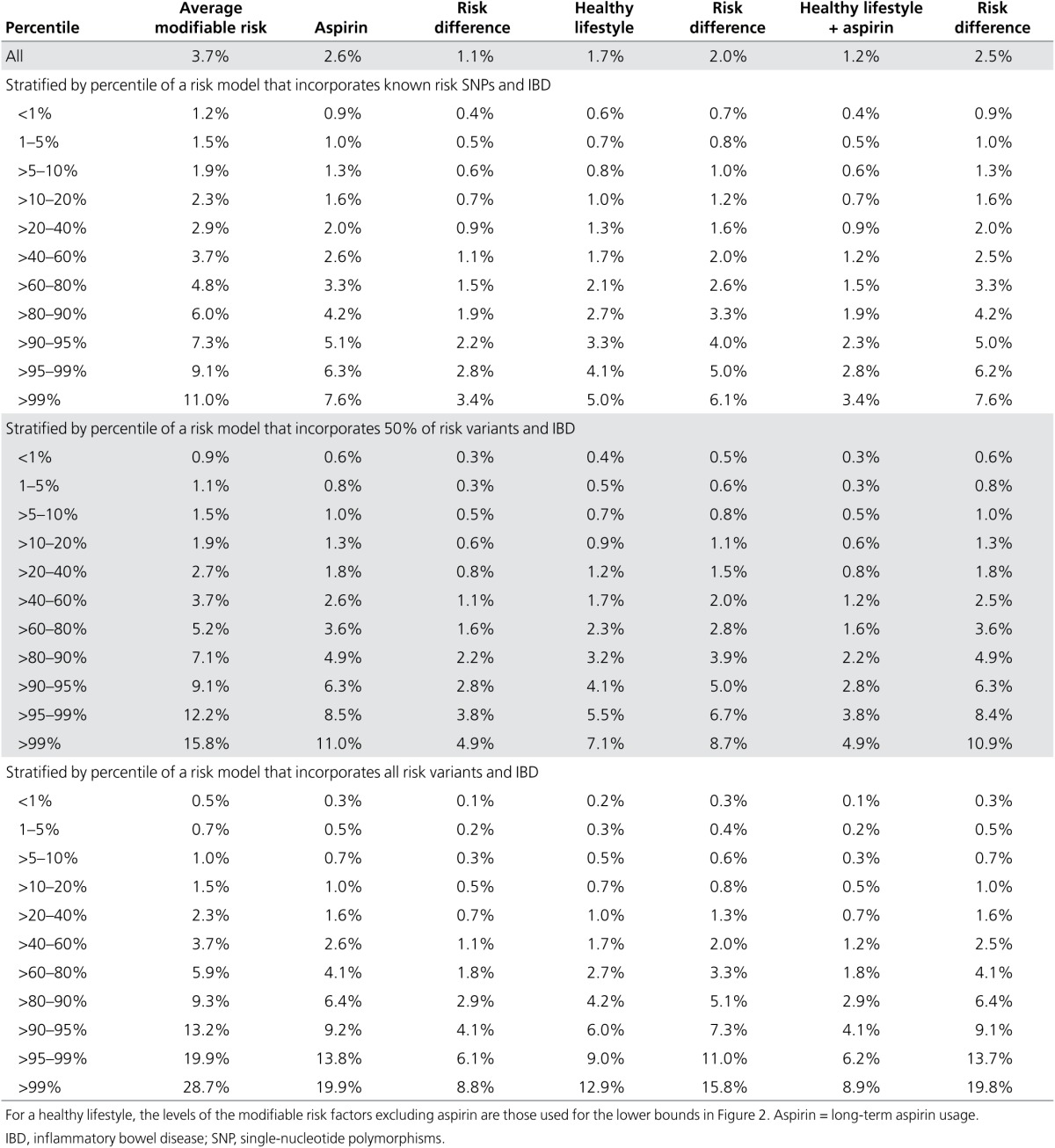
The 25-year absolute risk of developing colorectal cancer in 50-year-old men stratified by host risk factors
