# Supplementary material for: Modeling the prevention of colorectal cancer from the combined impact of host and behavioral risk factors
Source: Genet Med. 2016 Aug 4;19(3):314–21. doi: 10.1038/gim.2016.101 (PMC5133376; doi:10.1038/gim.2016.101)
Supplement: Supplementary Information [file gim2016101x1.zip › Supplementary Table S1.docx]

**Supplementary Table S1: Established common CRC susceptibility alleles.**

| **dbSNP ID** | **Locus** | **Gene** | **Risk-Allele Frequency** | **OR** | **Variance** | **Study** |
| --- | --- | --- | --- | --- | --- | --- |
| rs72647484 | 1p36.12 | *WNT4 - CDC42* | 0.91 | 1.24 | 0.005 | Al-Tassan 2015[1] |
| rs10911251 | 1q25.3 | *LAMC1* | 0.57 | 1.05 | 0.001 | Peters 2013[2] |
| rs6691170 | 1q41 | *DUSP10 - QRSL1P2* | 0.34 | 1.08 | 0.002 | Houlston 2010[3] |
| rs35360328 | 3p22.1 | *CTNNB1* | 0.16 | 1.14 | 0.003 | Schumacher 2015[4] |
| rs812481 | 3p14.1 | *LRIG1* | 0.58 | 1.09 | 0.002 | Schumacher 2015[4] |
| rs10936599 | 3q26.2 | *MYNN* | 0.76 | 1.08 | 0.002 | Houlston 2010[3] |
| rs35509282 | 4q32.2 | *FSTL5* | 0.09 | 1.53 | 0.02 | Schmit 2014[5] |
| rs647161 | 5q31.1 | *C5orf66* | 0.33 | 1.04 | 0.001 | Al-Tassan 2015[1] |
| rs1321311 | 6p21.31 | *TRNAI25* | 0.23 | 1.11 | 0.003 | Dunlop 2012[6] |
| rs16892766 | 8q23.3 | *LINC00536 - EIF3H* | 0.08 | 1.23 | 0.004 | Tomlinson 2008[7] |
| rs7014346 | 8q24.21 | *LOC101930033* | 0.65 | 1.19 | 0.009 | Tenesa 2008[8] |
| rs6983267 | 8q24.21 | *CCAT2;LOC101930033* | 0.49 | 1.18 | 0.009 | Tomlinson 2007[9] |
| rs10795668 | 10p14 | *RNA5SP299 - LINC00709* | 0.66 | 1.15 | 0.006 | Tomlinson 2008[7] |
| rs704017 | 10q22.3 | *ZMIZ1-AS1* | 0.56 | 1.07 | 0.002 | Al-Tassan 2015[1] |
| rs1035209 | 10q24.2 | *NKX2-3 - SLC25A28* | 0.19 | 1.15 | 0.004 | Whiffin 2014[10] |
| rs11190164 | 10q24.2 | *SLC25A28* | 0.29 | 1.09 | 0.002 | Schumacher 2015[4] |
| rs12241008 | 10q25 | *VTI1A* | 0.1 | 1.13 | 0.002 | Wang 2014[11] |
| rs174550 | 11q12.2 |  | 0.35 | 1.08 | 0.002 | Al-Tassan 2015[1] |
| rs3824999 | 11q13.4 | *POLD3* | 0.5 | 1.15 | 0.007 | Dunlop 2012[6] |
| rs3802842 | 11q23.1 | *COLCA2;COLCA1* | 0.68 | 1.14 | 0.005 | Tenesa 2008[8] |
| rs12309274 | 12p13.33 | *WNK1* | 0.14 | 1.07 | 0.001 | Al-Tassan 2015[1] |
| rs3217810 | 12p13.32 | *CCND2* | 0.12 | 1.07 | 0.001 | Peters 2013[2] |
| rs11169552 | 12q13.13 | *DIP2B - ATF1* | 0.74 | 1.1 | 0.002 | Houlston 2010[3] |
| rs3184504 | 12q24.12 | *SH2B3* | 0.53 | 1.09 | 0.002 | Schumacher 2015[4] |
| rs73208120 | 12q24.22 | *NOS1* | 0.11 | 1.16 | 0.003 | Schumacher 2015[4] |
| rs1957636 | 14q22.2 | *BMP4* | 0.6 | 1.09 | 0.002 | Tomlinson 2011[12] |
| rs4444235 | 14q22.3 | *RPS3AP46 - MIR5580* | 0.45 | 1.1 | 0.003 | Houlston 2008[13] |
| rs4779584 | 15q14 | *SCG5 - GREM1* | 0.82 | 1.18 | 0.006 | Tomlinson 2011[12] |
| rs9929218 | 16q22.1 | *CDH1* | 0.69 | 1.09 | 0.002 | Houlston 2008[13] |
| rs16941835 | 16q24.2 | *FOXL1* | 0.21 | 1.16 | 0.005 | Al-Tassan 2015[1] |
| rs4939827 | 18q21.2 | *SMAD7* | 0.52 | 1.22 | 0.013 | Broderick 2007[14] |
| rs10411210 | 19q12 | *RHPN2* | 0.91 | 1.12 | 0.001 | Houlston 2008[13] |
| rs1800469 | 19q13.2 |  | 0.31 | 1.07 | 0.001 | Al-Tassan 2015[1] |
| rs961253 | 20p12.3 | *FGFR3P3 - CASC20* | 0.37 | 1.11 | 0.004 | Houlston 2008[13] |
| rs4813802 | 20p12.3 | *BMP2* | 0.36 | 1.11 | 0.003 | Tomlinson 2011[12] |
| rs6066825 | 20q13.13 | *PREX1* | 0.64 | 1.09 | 0.002 | Schumacher 2015[4] |
| rs4925386 | 20q13.33 | *LAMA5* | 0.31 | 1.1 | 0.003 | Houlston 2010[3] |

**REFERENCES**

1. Al-Tassan NA, Whiffin N, Hosking FJ et al. A new GWAS and meta-analysis with 1000Genomes imputation identifies novel risk variants for colorectal cancer. Sci Rep 2015; 5: 10442.

2. Peters U, Jiao S, Schumacher FR et al. Identification of Genetic Susceptibility Loci for Colorectal Tumors in a Genome-Wide Meta-analysis. Gastroenterology 2013; 144: 799-807 e724.

3. Houlston RS, Cheadle J, Dobbins SE et al. Meta-analysis of three genome-wide association studies identifies susceptibility loci for colorectal cancer at 1q41, 3q26.2, 12q13.13 and 20q13.33. Nat Genet 2010; 42: 973-977.

4. Schumacher FR, Schmit SL, Jiao S et al. Genome-wide association study of colorectal cancer identifies six new susceptibility loci. Nat Commun 2015; 6: 7138.

5. Schmit SL, Schumacher FR, Edlund CK et al. A novel colorectal cancer risk locus at 4q32.2 identified from an international genome-wide association study. Carcinogenesis 2014; 35: 2512-2519.

6. Dunlop MG, Dobbins SE, Farrington SM et al. Common variation near CDKN1A, POLD3 and SHROOM2 influences colorectal cancer risk. Nat Genet 2012; 44: 770-776.

7. Tomlinson IP, Webb E, Carvajal-Carmona L et al. A genome-wide association study identifies colorectal cancer susceptibility loci on chromosomes 10p14 and 8q23.3. Nat Genet 2008; 40: 623-630.

8. Tenesa A, Farrington SM, Prendergast JG et al. Genome-wide association scan identifies a colorectal cancer susceptibility locus on 11q23 and replicates risk loci at 8q24 and 18q21. Nat Genet 2008; 40: 631-637.

9. Tomlinson I, Webb E, Carvajal-Carmona L et al. A genome-wide association scan of tag SNPs identifies a susceptibility variant for colorectal cancer at 8q24.21. Nat Genet 2007; 39: 984-988.

10. Whiffin N, Hosking FJ, Farrington SM et al. Identification of susceptibility loci for colorectal cancer in a genome-wide meta-analysis. Hum Mol Genet 2014; 23: 4729-4737.

11. Wang H, Burnett T, Kono S et al. Trans-ethnic genome-wide association study of colorectal cancer identifies a new susceptibility locus in VTI1A. Nat Commun 2014; 5: 4613.

12. Tomlinson IP, Carvajal-Carmona LG, Dobbins SE et al. Multiple common susceptibility variants near BMP pathway loci GREM1, BMP4, and BMP2 explain part of the missing heritability of colorectal cancer. PLoS Genet 2011; 7: e1002105.

13. Study C, Houlston RS, Webb E et al. Meta-analysis of genome-wide association data identifies four new susceptibility loci for colorectal cancer. Nat Genet 2008; 40: 1426-1435.

14. Broderick P, Carvajal-Carmona L, Pittman AM et al. A genome-wide association study shows that common alleles of SMAD7 influence colorectal cancer risk. Nat Genet 2007; 39: 1315-1317.
